# Delirium Severely Worsens Outcome in Patients with COVID-19—A Retrospective Cohort Study from Temporary Critical Care Hospitals

**DOI:** 10.3390/jcm10132974

**Published:** 2021-07-02

**Authors:** Katarzyna Kotfis, Wojciech Witkiewicz, Aleksandra Szylińska, Karina Witkiewicz, Magdalena Nalewajska, Wiktoria Feret, Łukasz Wojczyński, Łukasz Duda, Eugene Wesley Ely

**Affiliations:** 1 Department Anesthesiology, Intensive Therapy and Acute Intoxications, Pomeranian Medical University, 70-111 Szczecin, Poland; 2 Department of Cardiology, Pomeranian Medical University, 70-111 Szczecin, Poland; witkiewiczwojciech@gmail.com (W.W.); dudalukaszp@gmail.com (Ł.D.); 3 Department of Medical Rehabilitation and Clinical Physiotherapy, Pomeranian Medical University in Szczecin, 71-210 Szczecin, Poland; aleksandra.szylinska@pum.edu.pl; 4 Department of Pulmonology, Pomeranian Medical University, 70-891 Szczecin, Poland; karina.witkiewicz@gmail.com; 5 Department of Nephrology, Transplantology and Internal Medicine, Pomeranian Medical University, 70-111 Szczecin, Poland; nalewajska@gmail.com (M.N.); feretwiktoria@gmail.com (W.F.); wojczynski@gmail.com (Ł.W.); 6 Critical Illness Brain Dysfunction Survivorship Center, Nashville, Vanderbilt University Medical Center, Nashville, TN 37203, USA; wes.ely@vumc.org; 7 Division of Pulmonary and Critical Care Medicine, Department of Medicine, Vanderbilt University Medical Center, Nashville, TN 37232, USA; 8 Center for Health Services Research, Vanderbilt University Medical Center, Nashville, TN 37203, USA; 9 Geriatric Research, Education and Clinical Center Service, Department of Veterans Affairs Medical Center, Tennessee Valley Health Care System, Nashville, TN 37212, USA

**Keywords:** delirium, impaired consciousness, coronavirus, SARS-CoV-2, outcome, critical care, mortality

## Abstract

Delirium is a sign of deterioration of homeostasis and worse prognosis. The aim of this study was to investigate the frequency, risk factors and prognosis of delirium in patients with COVID-19 in a temporary acute setting hospital. A retrospective cohort analysis of data collected between October 2020 and February 2021 from two temporary acute care hospitals was performed. All consecutive hospitalized patients ≥18 years old with COVID-19 were included. An assessment of consciousness was carried out at least two times a day, including neurological examination. Delirium was identified through retrospective chart review according to DSM-5 criteria if present at least once during hospitalization. Analysis included 201 patients, 39 diagnosed with delirium (19.4%). Delirious patients were older (*p* < 0.001), frailer (*p* < 0.001) and the majority were male (*p* = 0.002). Respiratory parameters were worse in this group with higher oxygen flow (*p* = 0.013), lower PaO_2_ (*p* = 0.043) and higher FiO_2_ (*p* = 0.006). The mortality rate was significantly higher in patients with delirium (46.15% vs 3.70%, *p* < 0.001) with OR 17.212 (*p* < 0.001) corrected for age and gender. Delirious patients experienced significantly more complications: cardiovascular (OR 7.72, *p* < 0.001), pulmonary (OR 8.79, *p* < 0.001) or septic (OR 3.99, *p* = 0.029). The odds of mortality in patients with COVID-19 presenting with delirium at any point of hospitalization were seventeen times higher.

## 1. Introduction

Novel respiratory syndrome, known as COVID-19, was first reported at the end of December 2019 and soon became a global health threat [1]. With millions of people affected and suffering worldwide, the global burden for COVID-19 survivors is unprecedented. The COVID-19 pandemic introduced new criteria for immediate planning in healthcare to accommodate a rapidly growing number of admissions to critical care wards during the pandemic’s peak. Temporary hospitals were created to overcome the sudden surge of acutely ill and deteriorating patients into the intensive care units (ICUs) and toprovide professional medical help despite bed and resources shortages. The number of temporary acute care units introduced into the systems of all countries meant that the ability to care for many patients in a short time was higher, but on the other hand, also meant that the use of systematic patient assessments, using dedicated scales, might have been compromised [2].

Central nervous system (CNS) involvement during COVID-19 was identified early during the initial phase of the pandemic, therefore awareness of different aspects of the effects of SARS-CoV-2 virus on the central nervous system should be acknowledged by clinicians and scientists. A large observational study carried out by Mao et al. showed a high prevalence of central nervous system disorders, such as dizziness, headache, impaired consciousness, acute cerebrovascular disease, ataxia or presence of seizures [3]. Infection with SARS-CoV-2 causes acute brain dysfunction in the form of delirium in a significant proportion of patients in the acute stage of COVID-19 [4]. Data from the intensive care units reported that delirium prevalence ranged from 45% to 84%, depending on the delirium identification tools and definitions used in the studies [5,6,7]. Delirium is an acute brain disorder, potentially reversible, that commonly occurs in critically ill patients with a pathomechanism related to neuroinflammation and oxidative stress. Delirium identification is based on clinical observation and is characterized by rapid onset, significant symptom fluctuation during the day, disturbance of the wake and sleep cycle and changes in thinking, memory and behavior [8].

Undoubtedly, delirium monitoring should always be carried out in a formalized manner, using dedicated, well-established guidelines [9] and validated diagnostic scales, such as the Confusion Assessment Method for Intensive Care Unit (CAM-ICU) or the Intensive Care Delirium Screening Checklist (ICDSC) [10]. However, the adherence to clinical guidelines in temporary acute care hospitals, including delirium identification and management may have been and still may be low due to staff shortages and heavy workload [2]. In many places acutely ill patients were unable to receive a formal admission to the ICU and were provided with a bed at high-dependency acute units, which employed a wide range of non-ICU healthcare professionals, not trained in using validated scales to assess acute consciousness impairment.

In such a situation, formal monitoring of acute brain dysfunction within the temporary ICU is usually impossible to be implemented with the personnel turnover being too high and/or the willingness to implement an additional monitoring scale too low. The current COVID-19 pandemic has made it necessary to adapt the guidelines to real-life scenarios of a sudden increase in the number of patients over a short time [11,12]. The authors of this analysis focused on reporting real-life data regarding monitoring of acute changes of consciousness in a non-research, ad-hoc organized medical facility that had no training options for formal delirium screening. We hypothesized that it is crucial to identify delirium, as an early and often the only sign of deterioration of homeostasis (hypoxia, infection, ion disturbance, senses impairment or drug withdrawal) and treat accordingly. Therefore, the objective of this study was to investigate the frequency, risk factors and prognosis of delirium in patients with COVID-19 admitted to acute care temporary hospitals at the Pomeranian Medical University in Szczecin, Poland.

## 2. Materials and Methods

The authors conducted an observational retrospective cohort data analysis from 3 acute care units of 2 temporary hospitals in Szczecin, Poland. The data comes from an ad-hoc high-dependency acute care unit (“oxygen unit”) with passive oxygen, high-flow nasal oxygen therapy (HFNOT) and non-invasive ventilation (NIV). If any of the patients deteriorated, they were admitted to a temporary ICU (“ventilation unit”). The data was collected between October 2020 and February 2021, after receiving permission from the university hospital management.

### 2.1. Ethical Considerations

The study received a waiver from the Bioethical Committee of the Pomeranian Medical University due to its retrospective, observational nature (decision no. KB-0012/15/02/2021/Z dated 3 February 2021).

### 2.2. Study Population

Adult patients (≥18 years old) with positive antigen tests (approved in Poland with diagnostic sensitivity ≥90% and specificity ≥97%) or reverse transcription polymerase chain reaction (RT-PCR), hospitalized in the “oxygen unit” of temporary hospitals were included into the study. Authors excluded severely ill patients with predominant severe comorbidities without symptoms of coronavirus infection (especially pneumonia).

### 2.3. Data Collection

Data were retrieved from an electronic hospital database and included: study group characteristics (demographic data, comorbidities, addictions, medications on admission), COVID-19 symptoms on admission, laboratory testing on admission, respiratory and ventilation parameters, in-patient hospital treatment and complications. The assessment of consciousness was carried out at least 2 times a day, in the morning (between 9:00 and 11:00 a.m.) and in the evening (between 19:00 and 21:00 p.m.), including neurological examination and autopsychic and allopsychic orientation. Delirium was identified through a retrospective chart review method according to DSM-5 criteria if present at least once during hospitalization [7]. Data regarding outcome (death, hospital length of stay, ICU length of stay, complications, discharge information) were retrieved from the hospital computer database.

### 2.4. Statistical Analysis

All analyses were performed using licensed software Statistica 13 (StatSoft, Inc., Tulsa, OK, USA). The continuous variables are presented as mean, standard deviation (SD) and median. The categorical variables are presented as numbers and a percentage. The Mann–Whitney U test was used to compare continuous variables. The chi-square test or chi-square test with Yates’s correction was used to compare qualitative data between the two groups of patients. The receiver operating characteristic (ROC) curve analysis was performed to determine the best cut-off value for predicting NLR values for delirium prediction. Moreover, the analysis of the relationship between the complications, follow up and delirium was performed using logistic regression with model analysis adjusted by data (age and gender). Kaplan-Meier analysis calculated the probability of survival. Statistical significance was set at *p*-value ≤ 0.05.

## 3. Results

The analysis included a group of 201 patients, 162 did not develop signs of delirium during hospitalization, whilst 39/201 were diagnosed with at least one episode of delirium (19.4%). Demographic data and comorbidities on admission are presented in Table 1. Patients with delirium were more often males (*p* = 0.002), older (*p* < 0.001) and individuals who scored higher in the clinical frailty scale (CFS) (*p* < 0.001). The majority of patients with delirium had been previously diagnosed with arterial hypertension (*p* = 0.023), chronic heart failure (*p* = 0.019), chronic kidney disease (*p* = 0.048) and diabetes treated with insulin (*p* = 0.002).

Table 2 provides information on pre-admission medications used by the patients included in the study which were continued during hospitalization if no contraindications occurred. Individuals diagnosed with at least one delirium episode during hospitalization were treated with diuretics (*p* = 0.037) and insulin (*p* = 0.002) more often in comparison with non-delirious patients.

Individuals who were diagnosed with delirium during their hospital stay did not differ statistically from patients without consciousness impairment in terms of SARS-CoV-2-related symptoms upon admission to the hospital. The data are presented in Table 3.

Laboratory results on admission are visible in Table 4. There were significant differences between the two groups regarding the following parameters: higher leukocyte count (*p* = 0.001), neutrophil count (*p* < 0.001), neutrophil-to-lymphocyte ratio (NLR, *p* = 0.001), creatinine serum level (*p* = 0.001), urea serum level (*p* = 0.040), C-reactive protein serum level (CRP) (*p* = 0.018), interleukin-6 serum level (IL-6, *p* = 0.023), serum procalcitonin (*p* < 0.001), international normalized ratio (INR, *p* = 0.016), lactate dehydrogenase (LDH, *p* = 0.012), D-Dimer (*p* = 0.004), troponin T (TnT) (*p* = 0.005). The platelet-to-white blood cell ratio (PWR, *p* = 0.004) and glomerular filtration rate (GFR, *p* = 0.007) was lower in the delirium group. According to ROC analysis, the cut-off level for NLR was 6.51.

Ventilation parameters on admission between the two groups were compared and are visible in Table 5. The use of non-rebreather masks among patients with delirium was higher (*p* = 0.010) and the oxygen flow rates were higher in this group (*p* = 0.013). In patients with delirium, there were lower levels of partial pressure of oxygen (PaO_2_) in arterial blood gas samples when compared to non-delirious individuals (*p* = 0.043). Fraction of inspired oxygen (FiO_2_) rates were also higher in delirious patients (*p* = 0.006). A statistically significant higher percentage of delirium was observed in patients with assisted ventilation (HFNOT + NIV) than without this type of therapy (*p* < 0.001).

Table 6 presents the analysis of COVID-19-specific treatment administered during hospital stay. A greater percentage of patients with delirium required a therapeutic dose of low-molecular-weight heparin (LMWH) (*p* = 0.004). When assessing antibiotic therapy, a greater percentage of patients with delirium received treatment with azithromycin (*p* = 0.019) or another antibiotic other than ceftriaxone (*p* = 0.035). The analysis of steroid therapy showed that hydrocortisone was used in a greater number of patients with impaired consciousness (*p* < 0.001), and there was a higher maximum dose of dexamethasone (*p* = 0.037) among these individuals as well.

A detailed multivariable analysis of complications was performed. During treatment, a significantly greater number of delirious patients experienced cardiological (OR 7.720, *p* < 0.001), pulmonary (OR 8.788, *p* < 0.001) and sepsis (OR 3.991, *p* = 0.029). The mortality rate was higher in patients with delirium (46.15% vs. 3.70%, *p* < 0.001) with OR 17.212, *p* < 0.001). The data are presented in Table 7 and Table 8.

**Figure 1:**
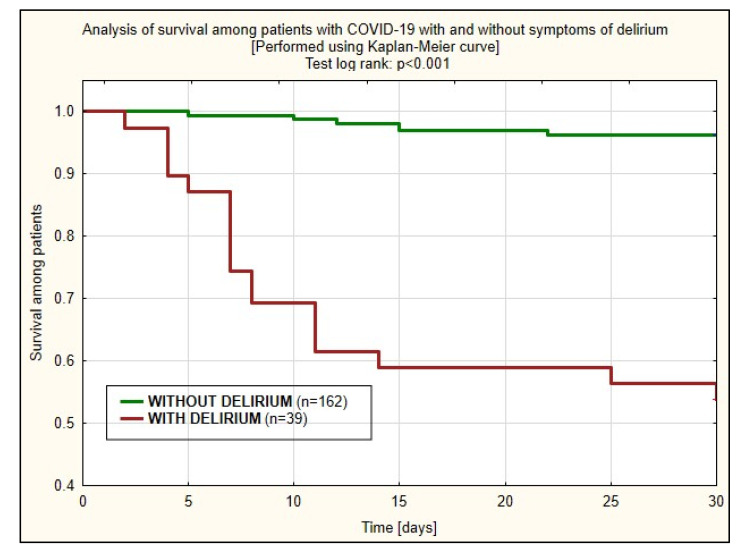
Analysis of survival among patients with COVID-19 with and without delirium.

A survival probability assessment in delirious and non-delirious COVID-19 patients was performed using the Kaplan-Meier curve (Figure 1). It presents a statistically significant difference in 30-day survival between both groups in favor of patients without delirium (*p* < 0.001).

## 4. Discussions

Delirium accounts for sudden, onset fluctuating impairment of consciousness commonly observed in critically ill patients that cannot be explained by preexisting neurological disorders [13,14]. The results of this study report that the odds of mortality in patients with COVID-19 presenting with delirium is seventeen times higher (as adjusted to age and gender) as compared to patients without this consciousness disturbance during hospital stay and reach an incidence rate of 46.15%. Delirium has been reported to contribute to worsened outcomes in severely ill patients, from prolonged hospitalization, to increased risk of long-term cognitive impairment, neuropsychiatric disorders and even death [15,16]. Much attention has been placed recently on the CNS complications of COVID-19, especially in patients presenting with severe infection [17] and in the elderly population [18]. Various factors could lead to delirium development in COVID-19. Direct pathological effects of the SARS-CoV-2 virus on the brain cells, release of CNS inflammatory mediators, peripheral organ systems insufficiency, sedative strategies, prolonged mechanical ventilation time and social isolation have been listed as possible contributing factors [12]. ARDS-related hypoxemia and oxidative stress are further possible underlying causes [19]. Other authors have also reported systemic infections, metabolic and endocrine alterations, such as electrolyte disbalance, anemia, hyperglycemia and hypoalbuminemia as etiologies related to delirium occurrence in COVID-19 [20].

Increased mortality in the delirium group had been previously confirmed in a study by Rebora et al. in an Italian cohort of COVID-19 patients hospitalized in four acute medical units [21]. Authors report that 14.1% of patients presented with delirium on admission displayed almost a two-fold chance of in-hospital mortality compared to those in the non-delirium group [21]. Our results show that 19% of patients were identified to have delirium, but mortality was 17-times higher. In a study by Kennedy et al., 28% of elderly patients with COVID-19 admitted to ED displayed signs of delirium at presentation [22]. In a retrospective study of COVID-19 patients performed by Mao et al., the incidence rate for impaired consciousness reached 14.8% in severe infections defined according to the American Thoracic Society guidelines for community-acquired pneumonia [2]. These are quite contrary to the results of a meta-analysis performed by Nazari et al., who reported impaired consciousness in 1.9% of COVID-19 patients [23]. In the study by Mao et al., patients more prone to develop consciousness disturbances were older and more burdened with chronic diseases, especially arterial hypertension, compared to the non-severe infection group [2]. These outcomes stay in accordance with our results as we indicate significantly increased mean age and increased prevalence of arterial hypertension in the group of patients with impaired consciousness. In our research, heart failure, chronic kidney disease and diabetes mellitus treated with insulin injections on admission also contributed to increased risk for altered states of consciousness during hospitalization. Interestingly, none of the comorbidities predominated in the delirium group in comparison to the non-delirium group in a cohort analysis of French ICU patients with COVID-19 [4].

We further report that the male sex and higher CFS scores predispose to delirium in a COVID-19 cohort. The male sex has been previously associated with disease severity measured on admission to the ICU and mortality in a global-scale meta-analysis of more than 3,000,000 cases [24]; however, to the best of our knowledge, no study has previously linked male sex to increased prevalence of consciousness disturbances. Frailty, defined as ≥ 5 points CFS was found to significantly predict delirium in a hospital cohort of elderly patients diagnosed with SARS-CoV-2 infection [25]. Similar conclusions have been drawn by Spanish researchers [26].

The intensity of oxygen therapy in the study group had a statistically significant link with the occurrence of delirium, although initial peripheral oxygen saturation did not differ between the two groups. As expected, higher oxygen flows, as well as the need to use non-rebreathing masks or assisted ventilation (both HFNOT and NIV), and hence high FiO_2_, were associated with greater risk of delirium (Table 5). These assumptions were confirmed by Pun et al. [6]. Nazari et al. and Krewulak et al. drew similar conclusions [23,27]. The implementation of mechanical ventilation was inextricably linked with the occurrence of ARDS, which in turn predisposes to hippocampus damage and cognitive impairment. It is simultaneously influenced by the level of hypoxia [28,29]. According to Krewulak et al., delirium itself is associated with worse outcomes and mechanical ventilation is a modifiable risk factor for impaired consciousness. All of the above has also been confirmed by our research. Therefore, any interventions aimed at avoiding the implementation of mechanical ventilation, or shortening its duration, can improve the prognosis. The results of our research show that the survival rate was largely influenced by pulmonary complications, including fibrosis. The risk of death increased almost 17-fold in the case of fibrotic lesions in the lung tissue in delirious patients.

According to Rello et al., the way oxygen therapy is administered in patients with COVID-19 is of great importance. Moderate or severe hypoxemia despite HFNOT usually requires intubation [30], worsening the prognosis. However, this thesis is not confirmed by the Frat study [31]. According to their data, in patients with non-hypercapnic acute hypoxemic respiratory failure, treatment with high-flow oxygen, standard oxygen or noninvasive ventilation did not result in significantly different intubation rates. There was a significant difference in favor of high-flow oxygen in 90-day mortality.

In our study, we also tried to discover any specific laboratory findings on admission that could act as predictors of developing consciousness impairment during COVID-19, as no symptoms at presentation were found to be significantly linked to an increased risk of mental deterioration. We found that higher inflammatory parameters (such as leukocyte and neutrophil count, CRP, IL-6, PCT or LDH) were observed in patients whose mental state had deteriorated during their hospital stay. McNeil et al. concluded that IL-6 serum level is independently associated with delirium prevalence and length but found no correlation with C-reactive protein levels [32]. Knopp et al. correspondingly to our findings, revealed that increased CRP and NLR (neutrophil-to-leukocyte ratio) were linked to worse outcomes [33]. Multiple studies have shown that hyperinflammation and cytokine storm may trigger neurological symptoms in COVID-19 patients but found no single specific biomarker that can be used as a predictor of deterioration. 

In our study, elevated creatinine and urea serum levels were linked to higher rates of cognition impairment. Interestingly, we found no significant difference in developing consciousness decline when it comes to ion composition on admission, but, as a human’s body can maintain homeostasis in a wide range, we speculate that biochemical signs of kidney injury may act as a herald here. The kidney can be a direct target for SARS-CoV-2 leading to tubular damage, therefore patients with AKI should be considered as those with a higher risk of complications, including neuropsychiatric manifestations and death [34,35,36]. In a study by Toklu et al., over 25% of COVID-19 patients with neurological symptoms, including altered mental status and impaired consciousness, had hypernatremia. In addition, 20% of this cohort had persistent hypernatremia for over 48 h of their hospital stay [37]. Maguire et al. found that impairment of cognition, elevation in CRP, urea and NLR, but decrease in lymphocyte count, were linked to increased 30-day mortality [38]. Another finding in our study seems to be somewhat intuitive: higher levels of serum D-dimer is linked with higher prevalence of delirium. Coagulation is known to be significantly altered in the course of SARS-CoV-2 infection, thus giving us a wide spectrum of clinical presentations, i.e., pulmonary embolism, stroke, arterial and venous thrombosis, excessive bleeding, etc. Diaz-Perez reported that acute alteration in mental status in COVID-19 patients can be the only presentation of multiple small foci of cerebral strokes [39]. Moreover, pulmonary embolism leading to hypoxia can be an underlying cause of consciousness impairment [40,41,42]. In our results as well, elevated levels of cardiac T-troponin were linked to higher occurrence of delirium during hospital stay. Imazio et al. hypothesized that during SARS-CoV-2 infection, myocardial injury has rather a non-ischemic character and occurs secondary to hypoxia, sepsis, embolism or myocarditis [43]. A meta-analysis by Sandoval et al. showed that in COVID-19 patients with elevated troponin serum levels, the mortality rate could reach up to 75% [44]. Having mentioned all of the above, we suggest that elevated serum troponin on admission could act as an early warning for delirium development and death.

The neutrophil-to-lymphocyte ratio (NLR) is a novel, non-specific index of disease severity, previously studied in a vast range of inflammatory morbidities or malignancies, and in delirium [45]. It has been previously reported that a higher NLR may be associated with delirium in severely ill patients with acute ischemic stroke (NLR > 4.86) [46], delirium in hospitalized elderly patients (NLR > 3.626) [47] and in postoperative delirium after hip fracture (NLR ≥ 3.5) [48]. There is no consensus on what a normal range of NLR is, as it seems to vary slightly depending on age and race. Luo et al. analyzed almost 6000 healthy Chinese adults and proposed that the NLR should fall between 0.88 and 4.0 [49]. In a study on 2212 healthy Iranians by Moosazadeh et al., the NLR normal value was established between 1.0 and 2.4 [50]. Christiansen et al., in a large register-based study, aimed to assess NLR distribution in the Danish population by comparing patients in general practice and ICU. They concluded that in the GP group with low CRP (“healthy”), the NLR median was 1.85, and that the ICU group had a significantly higher median NLR [51]. In our research, we studied the Polish population, in which normal reference ranges of NLR had not been evaluated yet. Nevertheless, we found a statistically significant difference between NLR values between non-delirious and delirious COVID-19 patients, the delirious patients presenting with a higher index, with the cut-off point at ≥6.51. To our knowledge this is the first study to report this phenomenon. 

We have considered short-term complications associated with COVID-19. They came from various systems, including cardiovascular, respiratory and urinary, with cardiological complications—mostly atrial fibrillation and heart failure—increasing the risk of consciousness disorders the most (by 7.7 times). Among pulmonary disorders, the strongest aggravating factor turned out to be fibrosis (risk increased 8 times), and to a lesser extent respiratory failure itself (6.3 times). The clinical manifestation of pneumonia was also worsening prognosis, as it was in the cases in a German report [52]. According to our results, sepsis increases the risk of developing mental impairment 4-fold. Despite the common knowledge about their link with worse prognosis in patients without COVID-19 [53,54], their interaction with SARS-CoV-2 infection and delirium is still not fully discovered and requires further research. To our knowledge, there is no available statistical data regarding this type of correlation. Many authors, including Völk [52] and Garcez [55], found one of the more frequent comorbidities, which were cerebrovascular diseases (ischemic stroke, TIA, etc.), which was not statistically significant in our research, despite the occurrence of such complications.

**Limitations:** The authors realize that this study is not without limitations. First, the retrospective character of the study should be acknowledged, rendering our ability to interpret the relationship between delirium and mortality as associative rather than cause and effect. The collected database reaches from 3 up to 7 months back. During that period some changes have been applied to the general guidelines of COVID-19 treatment (i.e., corticosteroids or antibiotics dosing), therefore its potential impact on the final prognosis in particular individuals might remain considerable. Second, the follow-up is terminated by the patient’s discharge from the hospital, so no further data on the survival or patient condition was collected subsequently. Third, the core of the staff of the temporary hospital was formed by clinicians of several specializations, principally representing subspecialties of internal medicine (nephrologists, cardiologists, pulmonologists), but sporadically also surgeons, neurologists and obstetricians. Whilst laboratory tests remain irrefutable, the approach to assessing delirium may vary amidst different specialists. The study was carried out in two medical centers, in which different emphasis was put on each of the aspects of diagnostics as well as treatment. Thus, a minor bias in data acquisition might have occurred. Fourth, according to critical care guidelines, dedicated scales should be used to identify delirium in acutely ill patients. Introducing the use of validated delirium screening scales was the greatest difficulty in a temporary hospital due to a rapid personnel changeover and lack of time to perform an additional task during the wave of admissions. Nevertheless, in everyday clinical practice with a heavy workload, even the simplest measures identifying impairment of consciousness will identify the patients at risk of having worse outcomes. Monitoring and prevention of delirium (hypoxia, infection, ion disturbance, senses impairment or drug withdrawal) is one available mechanism by which to attempt to reduce mortality. 

## 5. Conclusions

The results of this study report that the odds of mortality in patients with COVID-19 presenting with delirium during their hospital stay is over seventeen times higher as compared to patients without acute brain dysfunction. In critical illness, such as severe SARS-CoV-2 infection, delirium is an early and often the only sign of deterioration of homeostasis, and thus should be monitored and prevented to avoid increased mortality.

## Figures and Tables

**Table 1:** Baseline characteristics of patients included in the study.

Variables	Total	No Delirium (*n* = 162)	Delirium (*n* = 39)	*p*-Value
**Demographic data**				
Age [years], mean ± SD; Me	68.14 ± 13.82; 69.0	65.86 ± 13.65; 68.0	77.59 ± 10.13; 81.0	<0.001
Gender [male], *n* (%)	100 (49.75)	72 (44.44)	28 (71.79)	0.002
BMI [kg/m^2^], mean ± SD; Me	27.32 ± 5.21; 27.5	27.23 ± 5.33; 27.4	27.70 ± 4.69; 28.3	0.416
Smoking, *n* (%)	29 (14.43)	25 (15.82)	4 (10.26)	0.531
EF [%] (mean ± SD; Me)	47.22 ± 16.11; 55.0	51.00 ± 13.78; 55.0	28.33 ± 15.28; 25.0	0.051
CFS (1–9), (mean ± SD; Me)	3.57 ± 1.92; 3.0	3.14 ± 1.58; 3.0	5.41 ± 2.15; 6.0	<0.001
**Comorbidities**				
Arterial Hypertension, *n* (%)	131 (65.17)	99 (61.11)	32 (82.05)	0.023
Chronic Coronary Syndrome, *n* (%)	42 (20.89)	31 (19.14)	11 (28.21)	0.211
Myocardial Infarction, *n* (%)	22 (10.95)	18 (11.11)	4 (10.26)	0.878
Chronic Heart Failure, *n* (%)	36 (17.91)	24 (14.81)	12 (30.77)	0.019
Atrial Fibrillation, *n* (%)	37 (18.41)	27 (16.67)	10 (25.64)	0.194
Previous ischemic stroke, *n* (%)	15 (7.46)	10 (6.17)	5 (12.82)	0.281
Previous hemorrhagic stroke, *n* (%)	1 (0.49)	0 (0.00)	1 (2.63)	0.428
Transient Ischemic Attack, *n* (%)	2 (0.99)	1 (0.62)	1 (2.63)	0.841
CKD (GFR < 60) [mL/min/1.73 m^2^], *n* (%)	35 (17.41)	24 (14.81)	11 (28.21)	0.048
Post-renal transplant, *n* (%)	4 (1.99)	4 (2.47)	0 (0.00)	0.724
Dialysis, *n* (%)	4 (1.99)	4 (2.47)	0 (0.00)	0.724
Impaired insulin tolerance, *n* (%)	5 (2.49)	4 (2.47)	1 (2.63)	0.590
Diabetes [oral medications/diet], *n* (%)	39 (19.40)	32 (19.75)	7 (17.95)	0.976
Diabetes [insulin], *n* (%)	23 (11.44)	13 (8.02)	10 (25.64)	0.002
Gout/hyperuricemia, *n* (%)	11 (5.47)	6 (3.70)	5 (12.82)	0.064
ICA stenosis, *n* (%)	10 (4.97)	6 (3.70)	4 (10.26)	0.201
Chronic peripheral ischemia, *n* (%)	11 (5.47)	7 (4.32)	4 (10.26)	0.284
Venous thrombosis, *n* (%)	4 (1.99)	2 (1.35)	2 (5.13)	0.408
Pulmonary Embolism, *n* (%)	2 (0.99)	2 (1.35)	0 (0.00)	0.885
COPD, *n* (%)	14 (6.97)	9 (5.56)	5 (12.82)	0.211
Asthma, *n* (%)	16 (7.96)	12 (7.41)	4 (10.26)	0.794
Active neoplasm, *n* (%)	17 (8.46)	15 (.26)	2 (5.13)	0.609

Legend: COPD—chronic obstructive pulmonary disease, CPI—chronic peripheral ischemia, Me—median, BMI—body mass index, CFS—clinical frailty scale, CKD—chronic kidney disease, EF—ejection fraction, GFR—glomerular filtration rate, ICA—internal carotid artery, *n*—number of patients, *p*—statistical significance, SD—standard deviation.

**Table 2:** Regular medications taken by the patient prior to admission.

Medications	No Delirium (*n* = 162)	Delirium (*n* = 39)	*p*-Value
ASA, *n* (%)	36 (22.22)	13 (34.21)	0.181
ADP inhibitors, *n* (%)	11 (6.83)	0 (0.00)	0.207
OAC/NOAC, *n* (%)	19 (11.80)	5 (13.16)	0.963
B-blockers, *n* (%)	78 (48.45)	22 (57.89)	0.386
ACE-I/Sartans, *n* (%)	71 (44.10)	14 (36.84)	0.523
CCBs, *n* (%)	34 (21.12)	9 (23.68)	0.899
Statins/fibrates, *n* (%)	42 (26.09)	10 (26.32)	0.860
Nitrates, *n* (%)	3 (1.86)	1 (2.63)	0.735
Diuretics, *n* (%)	53 (32.92)	20 (52.63)	0.037
MCRAsm, *n* (%)	13 (8.07)	5 (13.16)	0.504
Bronchodilators, *n* (%)	19 (11.80)	3 (7.89)	0.687
Oral antidiabetic drugs, *n* (%)	33 (20.50)	8 (21.05)	0.883
Insulin, *n* (%)	13 (8.07)	10 (26.32)	0.002
Thyroid hormones/thyrostatics, *n* (%)	24 (14.91)	3 (7.89)	0.383
NSAIDs, *n* (%)	9 (5.59)	0 (0.00)	0.290
Immunosuppression, *n* (%)	11 (6.83)	0 (0.00)	0.207
Opioids, *n* (%)	2 (1.24)	4 (10.53)	0.013

Legend: ASA—acetylsalicylic acid, ADP inhibitors—adenosine diphosphate receptor inhibitors, OAC—oral anticoagulants, NOAC—new oral anticoagulants, ACE-I—angiotensin-converting enzyme inhibitor, CCBs—calcium channel blockers, MCRAs—mineralocorticoid receptor antagonists, *n*—number of patients, NSAIDs—nonsteroidal anti-inflammatory drugs, *p*—statistical significance.

**Table 3:** Coronavirus-related symptoms on admission to the hospital.

Symptoms on Admission	No Delirium (*n* = 162)	Delirium (*n* = 39)	*p*-Value
Low-grade fever/Fever, *n* (%)	101 (62.35)	25 (64.10)	0.838
Dyspnea, *n* (%)	88 (54.32)	24 (61.54)	0.525
Cough, *n* (%)	79 (48.77)	13 (33.33)	0.119
Chest pain, *n* (%)	27 (16.67)	6 (15.38)	0.963
Weakness, *n* (%)	110 (67.90)	32 (82.05)	0.122
Nausea, *n* (%)	22 (13.58)	2 (5.13)	0.235
Vomiting, *n* (%)	19 (11.73)	1 (2.56)	0.156
Diarrhea, *n* (%)	26 (16.05)	2 (5.13)	0.131
Musculo-articular pains, *n* (%)	23 (14.20)	3 (7.69)	0.412
Lack of taste, *n* (%)	17 (10.49)	3 (7.69)	0.821
Lack of smell, *n* (%)	15 (9.26)	1 (2.56)	0.290
Headache, *n* (%)	13 (8.02)	1 (2.56)	0.394

Legend: *n*—number of patients, *p*—statistical significance.

**Table 4:** Laboratory results on admission.

Laboratory Data on Admission	No Delirium (*n* = 162)	Delirium (*n* = 39)	*p*-Value
	Mean ± SD; Me	Mean ± SD; Me	
HbA_1c_ (%)	6.73 ± 1.40; 6.2	6.68 ± 1.82; 5.90	0.495
TC [mg/dL]	147.91 ± 51.88; 137.0	153.0 ± 55.26; 135.0	0.716
LDL [mg/dL]	84.82 ± 39.31; 85.5	83.90 ± 38.87; 70.5	0.900
HDL [mg/dL]	36.38 ± 12.43; 33.5	39.50 ± 21.31; 33.5	0.922
TG [mg/dL]	159.09 ± 116.90; 124.5	144.30 ± 71.32; 133.0	0.989
WBC [10^9^/L]	6.94 ± 3.92; 6.0	9.85 ± 5.56; 9.1	0.001
Neutrophils [10^9^/L]	5.10 ± 3.27; 4.2	8.15 ± 5.37; 7.6	<0.001
Lymphocytes [10^9^/L]	1.17 ± 0.78; 1.0	1.03 ± 0.41; 1.1	0.984
NLR	5.75 ± 4.91; 4.5	9.25 ± 6.81; 6.9	0.001
NLR ≥ 6.51, *n* (%)	38 (27.94%)	22 (61.11%)	<0.001
PLT [10^9^/L]	241.85 ± 118.89; 214.0	255.42 ± 118.40; 239.0	0.420
PWR	39.76 ± 18.48; 36.6	30.93 ± 16.58; 29.2	0.004
PLR	263.70 ± 183.03; 227.22	280.95 ± 170.56; 220.6	0.314
HGB [mmol/L]	7.82 ± 1.50; 8.0	8.09 ± 1.49; 8.0	0.548
HCT [L/L]	0.36 ± 0.07; 0.4	0.38 ± 0.05; 0.4	0.199
Creatinine [mg/dL]	1.17 ± 1.07; 0.9	1.83 ± 1.94; 1.2	0.001
GFR [mL/min/1.73 m^2^]	76.73 ± 32.07; 75.2	60.91 ± 35.90; 53.9	0.007
Urea [mg/dL]	49.35 ± 30.69; 40.0	85.22 ± 75.43; 54.1	0.040
CRP [mg/dL]	69.59 ± 57.68; 55.0	98.31 ± 74.30; 81.1	0.018
IL-6 [pg/mL]	57.30 ± 76.69; 37.4	266.43 ± 1005.49; 57.9	0.023
PCT [ng/mL]	0.56 ± 2.89; 0.1	4.80 ± 18.30; 0.2	<0.001
AST [U/L]	46.54 ± 54.40; 32.5	64.32 ± 64.72; 47.0	0.163
Alanine transaminase (ALT) [U/L]	45.90 ± 79.82; 24.0	37.39 ± 29.91; 28.0	0.892
GGTP [U/L]	71.60 ± 77.21; 38.0	81.65 ± 166.66; 40.0	0.766
APRI	0.34 ± 1.15; 0.17	0.33 ± 0.48; 0.17	0.326
INR	1.30 ± 1.03; 1.1	1.78 ± 2.25; 1.2	0.016
APTT [s]	33.59 ± 9.06; 30.8	32.47 ± 7.42; 31.6	0.980
LDH [U/L]	322.42 ± 109.70; 308.0	427.13 ± 183.54; 346.0	0.012
Fibrinogen [g/L]	4.69 ± 1.65; 4.7	4.63 ± 2.28; 3.8	0.590
D-Dimer [ng/mL]	1753.61 ± 2229.58; 833.0	2953.18 ± 2779.1; 1518.0	0.004
CKMB [U/L]	20.37 ± 10.98; 18.0	25.88 ± 17.27; 21.3	0.099
TnT [ug/L]	0.07 ± 0.30; 0.01	0.05 ± 0.06; 0.03	0.005
Kalium [mmol/L]	4.08 ± 0.56; 4.1	4.23 ± 0.70; 4.21	0.305
Natrium [mmol/L]	136.10 ± 4.88; 137.0	137.72 ± 5.53; 138.0	0.157
Chloride [mmol/L]	98.46 ± 6.04; 99.0	99.70 ± 4.6; 100.0	0.482

Legend: ALT—alanine transaminase, APRI—AST-to-platelet ratio index, APTT—activated partial thromboplastin time, AST—aspartate transaminase, CK-MB—creatine kinase type MB, CRP—C-reactive protein, GFR—glomerular filtration rate, GGTP—gamma-glutamyl transferase, HbA1C—glycated hemoglobin, HCT—hematocrit, HDL—high-density lipoprotein, HGB—hemoglobin, Il-6—interleukin 6, INR—international normalized ratio, LDH—lactate dehydrogenase, LDL—low-density lipoprotein, Me—median, *n*—number of patients, NLR—neutrophil-to-lymphocyte ratio, *p*—statistical significance, TC—total cholesterol, TG—triglyceride, PCT—procalcitonin, PLR—platelet-to-lymphocyte ratio, PLT—platelets, PWR—platelet-to-WBC ratio, SD—standard deviation, TnT—troponin T, WBC—white blood cells.

**Table 5:** Respiratory parameters on admission.

Respiratory Parameters on Admission		No Delirium (*n* = 162)	Delirium (*n* = 39)	*p*-Value
SpO_2_ (mean ± SD; Me)		94.85 ± 3.17; 95.0	94.26 ± 4.46; 95.0	0.668
Nasal cannula, *n* (%)		51 (31.68%)	15 (38.46%)	0.419
Non-rebreather mask, *n* (%)		20 (12.42%)	12 (30.77%)	0.010
HFNOT	Yes, *n* (%)	6 (3.7 %)	9 (23.08 %)	<0.001
	Day started (mean ± SD; Me)	5.00 ± 3.95; 3.5	3.13 ± 3.00; 2.5	0.272
Flow [L/min] (mean ± SD; Me)		6.02 ± 3.83; 5.0	10.04 ± 8.68; 7.0	0.013
pH (mean ± SD; Me)		7.47 ± 0.06; 7.5	7.48 ± 0.06; 7.5	0.588
pO_2_ (mmHg), (mean ± SD; Me)		76.05 ± 24.06; 71.0	70.72 ± 29.72; 62.0	0.043
pCO_2_ (mmHg), (mean ± SD; Me)		34.66 ± 6.72; 34.0	32.04 ± 5.56; 31.0	0.105
FiO_2_ (mean ± SD; Me)		0.45 ± 0.29; 0.3	0.63 ± 0.28; 0.8	0.006
HCO_3_^-^ (mean ± SD; Me)		25.25 ± 4.87; 25.1	25.11 ± 5.28; 25.2	0.907
BE (mean ± SD; Me)		1.89 ± 4.62; 1.9	1.26 ± 5.76; 0.4	0.466
pO_2_/FiO_2_, (mean ± SD; Me)		252.89 ± 161.44; 26.36	158.45 ± 134.38; 82.7	0.005
ARDS, *n* (%)	without	25 (39.68%)	4 (16.00%)	0.029
	mild	11 (17.46%)	2 (8.00%)	
	moderate	11 (17.46%)	5 (20.00%)	
	severe	16 (25.40%)	14 (56.00%)	

Legend: ARDS—acute respiratory distress syndrome, BE—base excess, FiO_2_—fraction of inspired oxygen, HFNOT—high-flow nasal oxygen therapy, Me—median, *n*—number of patients, *p*—statistical significance, pCO_2_—partial pressure of carbon dioxide, pO_2_—partial pressure of oxygen, SD—standard deviation, SpO_2_—peripheral oxygen saturation.

**Table 6:** Data regarding COVID-19-specific treatment during hospitalization.

COVID-19-Specific Treatment	No Delirium (*n* = 162)	Delirium (*n* = 39)	*p*-Value
**LMWH, *n* (%)**	**152 (93.83%)**	**39 (100.00%)**	**0.111**
Prophylactic dose [40 mg once a day], *n* (%)	68 (41.98%)	11(28.21%)	0.114
Intermediate dose [1 mg/kg once a day], *n* (%)	60 (37.04%)	13 (33.33%)	0.805
Therapeutic dose [1 mg/kg twice a day], *n* (%)	39 (24.07%)	19 (48.72%)	0.004
**Antibiotic therapy, *n* (%)**	**145 (89.51%)**	**37 (94.87%)**	**0.469**
Ceftriaxone, *n* (%)	127 (78.40%)	35(89.74%)	0.167
Azithromycin, *n* (%)	108 (66.67%)	34 (87.18%)	0.019
Levofloxacin, *n* (%)	14 (8.64%)	3 (7.69%)	0.897
Other antibiotic, *n* (%)	26 (16.05%)	12 (30.77%)	0.035
**Steroid therapy, *n* (%)**	**113 (69.75%)**	**32 (82.05%)**	**0.181**
Dexamethasone, *n* (%)	108 (66.67%)	30 (76.92%)	0.295
Prednisone, *n* (%)	5 (3.09%)	0 (0.00%)	0.590
Hydrocortisone, *n* (%)	1 (0.62%)	6 (15.38%)	<0.001
Other steroid, *n* (%)	1 (0.62%)	1 (2.56%)	0.841
Max. dexamethasone dose (or equivalent) (mean ± SD; Me)	6.19 ± 3.04; 4.0	13.41 ± 34.20; 8.0	0.037
Time of steroid therapy [days] (mean ± SD; Me)	8.16 ± 4.91; 7.0	8.00 ± 6.70; 7.0	0.377
**Vitamin D3, *n* (%)**	**41 (25.31%)**	**11 (28.21%)**	**0.867**
**Remdesivir, *n* (%)**	**31 (19.14%)**	**5 (12.82%)**	**0.489**

Legend: LMWH—low molecular weight heparin, Me—median, *n*—number of patients, *p*—statistical significance, SD—standard deviation.

**Table 7:** Complications and follow-up in COVID-19 patients with and without delirium.

Complications		No Delirium (*n* = 162)	Delirium (*n* = 39)	*p*-Value
**Cardiological complications (*n*/%)**		**10 (6.17%)**	**14 (35.90%)**	**<0.001**
Heart Failure, *n* (%)		5 (3.09%)	5 (12.82%)	0.036
Myocardial Infarction, *n* (%)		1 (0.62%)	0 (0.00%)	0.438
Atrial Fibrillation, *n* (%)		7 (4.32%)	7 (17.95%)	0.008
Atrial Flutter, *n* (%)		0 (0.00%)	1 (2.56%)	0.438
Other arrhythmias (including ventricular, supraventricular arrhythmias and atrioventricular conduction disorders), *n* (%)		2 (1.23%)	0 (0.00%)	0.841
**Pulmonary complications, *n* (%)**		**105 (64.81%)**	**35 (89.74%)**	**0.004**
Respiratory failure (pO_2_ < 60 mmHg and/or pCO_2_ > 45 mmHg), *n* (%)		23 (14.20%)	23 (58.97%)	<0.001
Radiological signs of pneumonia, *n* (%)		136 (84.47%)	37 (94.87%)	0.148
Clinical manifestation of pneumonia, *n* (%)		117 (72.22%)	35 (89.74%)	0.038
Fibrosis, *n* (%)		2 (1.24%)	7 (17.95%)	<0.001
Pneumothorax, *n* (%)		2 (1.24%)	1 (2.56%)	0.901
Hydrothorax, *n* (%)		15 (9.32%)	7 (17.95%)	0.207
**Renal complications, *n* (%)**		**22 (13.58%)**	**13 (33.33%)**	**0.004**
AKI or decompensation of CKD (creatinine level ratio (last measurement/admission)), *n* (%)		21 (12.96%)	12 (30.77%)	0.014
Urinary Tract Infection, *n* (%)		12 (7.41%)	7 (17.95%)	0.086
**Neurological complications, *n* (%)**		**0 (0.00%)**	**30 (76.92%)**	**<0.001**
Transient Ischemic Attack, *n* (%)		0 (0.00%)	1 (2.56%)	0.438
Ischemic stroke, *n* (%)		0 (0.00%)	1 (2.56%)	0.438
Seizures, *n* (%)		1 (0.62%)	0 (0.00%)	0.438
**Venous Thromboembolism, *n* (%)**		**3 (1.85%)**	**3 (7.69%)**	**0.161**
Deep Vein Thrombosis, *n* (%)		1 (0.62%)	0 (0.00%)	0.438
Pulmonary Embolism, *n* (%)		3 (1.85%)	3 (7.69%)	0.161
**Other complications, *n* (%)**		12 (7.41%)	8 (20.51%)	0.031
Pressure ulcers, *n* (%)		2 (1.23%)	3 (7.69%)	0.079
Gastrointestinal hemorrhage, *n* (%)		3 (1.85%)	2 (5.13%)	0.544
Mucosal bleeding, *n* (%)		3 (1.85%)	1 (2.56%)	0.724
HIT, *n* (%)		0 (0.00%)	1 (2.56%)	0.438
Sepsis, *n* (%)		8 (4.94%)	7 (17.95%)	0.014
CDI, *n* (%)		4 (2.47%)	0 (0.00%)	0.724
**FOLLOW-UP**				
Time of stay in the ward (including the day of admission and discharge) (mean ± SD; Me)		10.48 ± 5.22; 10.0	11.46 ± 9.10; 9.0	0.588
Timing of death (until day 30)		13.17 ± 5.71; 13.5	9.56 ± 7.24; 7.0	0.096
**Death, *n* (%)**		**6 (3.70%)**	**18 (46.15%)**	**<0.001**
Discharge, *n* (%)	Discharged home	136 (83.95%)	14 (35.90%)	<0.001
	Transferred to another unit	12 (7.41%)	1 (2.56%)	
	Transferred to ICU	8 (4.94%)	6 (15.38%)	

Legend: AKI—acute kidney injury, CDI—*Clostridium difficile* infection, CKD—chronic kidney disease, EF—ejection fraction, HIT—heparin-induced thrombocytopenia, ICU—intensive care unit, Me—median, *n*—number of patients, *p*—statistical significance, pCO_2_—partial pressure of carbon dioxide, pO_2_—partial pressure of oxygen, PE—pulmonary embolism, SD—standard deviation, TIA—transient ischemic attack.

**Table 8:** Logistic regression for patients with impaired consciousness.

Complications	Delirium(Unadjusted)		Delirium *(Adjusted by Age and Gender)	
	OR	*p*-Value	OR	*p*-Value
**Cardiological complications**	8.512 (3.409–21.256)	<0.001	7.720 (2.668–22.335)	<0.001
Heart Failure	4.618 (1.266–16.840)	0.020	4.449 (1.090–18.160)	0.038
Atrial Fibrillation	4.844 (1.589–14.766)	0.006	3.112 (0.886–10.930)	0.077
**Pulmonary complications**	4.750 (1.607–14.037)	0.005	8.788 (2.604–29.661)	<0.001
Respiratory failure (pO_2_ < 60 mmHg and/or pCO_2_ > 45 mmHg)	8.687 (3.999–18.871)	<0.001	6.285 (2.657–14.865)	<0.001
Radiological signs of pneumonia	3.401 (0.770–15.021)	0.106	3.812 (0.792–18.356)	0.095
Clinical manifestation of pneumonia	3.365 (1.131–10.011)	0.029	3.921 (1.187–12.957)	0.025
Pulmonary Fibrosis	17.391 (3.453–87.589)	<0.001	8.124 (1.458–45.271)	0.017
Pneumothorax	2.092 (0.185–23.680)	0.551	2.763 (0.225–33.987)	0.427
Hydrothorax	2.129 (0.803–5.647)	0.129	1.366 (0.451–4.132)	0.581
**Renal complications**	3.182 (1.425–7.105)	0.005	2.411 (0.945–6.149)	0.065
AKI/Decompensation of CKD (creatinine level ratio (last measurement/admission)	2.984 (1.314–6.776)	0.009	1.665 (0.649–4.268)	0.289
Urinary Tract Infection	2.734 (0.999–7.487)	0.050	2.602 (0.767–8.824)	0.125
**Pulmonary Embolism**	**4.417 (0.856–22.785)**	**0.076**	3.231 (0.458–22.774)	0.239
**Other complications**	3.226 (1.217–8.549)	0.019	2.321 (0.739–7.291)	0.149
Pressure ulcers	6.667 (1.074–41.368)	0.042	3.525 (0.469–26.519)	0.221
Gastrointestinal hemorrhage	2.865 (0.462–17.764)	0.258	1.766 (0.257–12.137)	0.563
Mucosal bleeding	1.395 (0.141–13.783)	0.776	1.855 (0.162–21.194)	0.619
Sepsis	4.211 (1.425–12.443)	0.009	3.991 (1.151–13.841)	0.029
**FOLLOW-UP**				
Time of stay in ward (including the day of admission and discharge)	1.024 (0.971–1.081)	0.374	1.009 (0.951–1.070)	0.766
Timing of death (until day 30)	0.931 (0.819–1.060)	0.279	0.913 (0.772–1.081)	0.291
**Death**	22.286 (7.955–62.434)	<0.001	17.212 (5.108–58.003)	<0.001

Legend: AKI—acute kidney injury, CKD—chronic kidney disease, *n*—number of patients, OR—odds ratio, *p*—statistical significance, pCO_2_—partial pressure of carbon dioxide, pO_2_—partial pressure of oxygen. Note: Delirium * adjusted by age and gender.

## Data Availability

Data will be available upon request.
